# Genome-Wide Microsatellite Characterization and Molecular Marker Development of Himalayan Griffon (*Gyps himalayensis*)

**DOI:** 10.3390/ani15101438

**Published:** 2025-05-16

**Authors:** Weibin Guo, Dianhua Ke, Changcao Wang, Haiying Fan

**Affiliations:** 1 School of Life Sciences, Jinggangshan University, Ji’an 343009, China; guowb1212@163.com (W.G.); ssk002whu@163.com (D.K.); 2 State Key Laboratory of Biocatalysis and Enzyme Engineering, School of Life Sciences, Hubei University, Wuhan 430062, China; wchangcao@163.com

**Keywords:** avian scavengers, vultures, genomic SSRs, polymorphism, molecular marker

## Abstract

The Himalayan griffon (*Gyps himalayensis*), an obligate scavenging bird in the Qinghai–Tibet Plateau, provides significant ecological services but is currently listed as near threatened. Despite their importance for conservation, genome-wide screening for microsatellites (or SSRs) in *G. himalayensis* and the development of corresponding molecular markers have been lacking. This study presents the first in-depth characterization of genome-wide microsatellites in *G. himalayensis* and the successful development of polymorphic SSR markers. A total of 240,741 microsatellite loci were detected in the *G. himalayensis* genome, with an average density of 202.2 SSRs per Mb, accounting for 0.44% of the genome. Mononucleotide repeats (53.2%) were the most prevalent among the different microsatellite motif types. Additionally, 100 primer pairs were initially identified, with 17 found to be polymorphic markers. These loci hold significant potential for revealing genotypic diversity in *G. himalayensis*, thereby laying a foundation for the conservation of this species.

## Simple Summary

The Himalayan griffon (*Gyps himalayensis*), a near-threatened scavenger in the Qinghai–Tibet Plateau, lacks genome-wide SSR markers for conservation studies. This research characterized microsatellites in its genome, identifying 240,741 SSRs (202.2 per Mb), with mononucleotide repeats (53.2%) being the most common. Seventeen polymorphic SSR markers were developed from 100 primer pairs, enabling genotypic diversity assessment. These findings provide essential tools for *G. himalayensis* conservation.

## 1. Introduction

The Himalayan griffon (*Gyps himalayensis*), often referred to as “nature’s clean-up crew”, is an obligate scavenging bird of prey primarily found in the Qinghai–Tibet Plateau. By consuming carrion, Himalayan griffons effectively mitigate disease transmission risks [1,2], thereby delivering essential ecosystem services that benefit both human populations and wildlife communities. These scavengers also carry profound cultural symbolism in plateau regions, serving as embodiments of spiritual metamorphosis through their carrion consumption, which mediates biospheric transitions from decay to renewal. Despite their ecological and cultural importance, Himalayan griffons face threats such as habitat loss, poisoning from ingesting toxic substances, and human persecution [3], leading to their classification as near threatened (BirdLife International, Cambridge, UK, 2022). This underscores the urgent need for conservation efforts to protect this species.

Conservation efforts for species like the Himalayan griffon must focus on preserving genetic diversity, which is crucial for population survival and adaptation [4,5,6]. Among molecular tools, microsatellites (simple sequence repeats—SSRs) have become indispensable for assessing genetic diversity due to their random and wide distribution in the genome, high polymorphism, codominant inheritance, ease of amplification, and low DNA quality requirements [7]. These attributes establish SSRs as invaluable markers for genetic diversity studies [8,9,10,11], whereas their genome-wide dispersion patterns underscore the crucial roles in genome evolution and phenotypic adaptation [12,13,14].

However, the species-specific nature of microsatellites requires customized primer development for PCR amplification and detection. Conventional approaches for developing microsatellite primers, such as genome enrichment libraries, cloning, and sequencing, are time-consuming, expensive, and inefficient. Furthermore, the low genomic density of these repetitive elements in some species creates technical challenges that restrict their utility [7,15,16]. Recent advancements in high-throughput genome sequencing have enabled the development of genome-wide polymorphic SSR markers for non-model organisms. By leveraging genome sequence information and bioinformatics tools, researchers can now identify and design primers for microsatellites more efficiently [17,18,19,20,21,22,23,24]. These genomic methods significantly reduce the time and cost required for primer development and provide valuable insights into both the abundance and distribution of microsatellites, which contributes to population genetics and functional genomics research.

This study aims to investigate the abundance and distribution characteristics of microsatellites in the Himalayan griffon genome by developing and identifying microsatellite primers, laying the foundation for the study and conservation of the genetic diversity of the Himalayan griffon.

## 2. Materials and Methods

### 2.1. Simple Sequence Repeats Mining in the Genome of Himalayan Griffon

The Himalayan griffon genome in FASTA format was downloaded from the National Center for Biotechnology Information (NCBI) database, and genomic SSRs were identified using the SciRoKo 3.4 program. SciRoKo offers a user-friendly interface and integrates functions for microsatellite classification and simple statistics [25]. Parameters were set to a minimum score of 15 and a mismatch penalty of 5 to search for mono-, di-, tri-, tetra-, penta-, and hexanucleotide repeats.

### 2.2. Feather Sampling and DNA Extraction

Molted feathers are a valuable source of DNA for protected avian species. Shed feathers were collected from celestial burial platforms at Sera Temple, Zhigongti Temple, and Kangba Temple in the Tibet Autonomous Region in August 2024. Feathers were stored in plastic bags at room temperature under dry-and-dark conditions for about two weeks before being transferred to −20 °C. Primary and secondary feathers were primarily collected due to their larger size and good blood supply during growth, which ensured sufficient DNA content. DNA was extracted from the basal tip of the calamus and/or the superior umbilicus using the Universal DNA Extraction Kit (TSINGKE, Beijing, China). Ultimately, DNA was successfully obtained from 52 individual samples.

### 2.3. SSR Primer Design

Primer3 v.2.6.1 was used to design primers flanking the identified microsatellite regions. The software considers factors such as primer length, melting temperature (Tm), GC content, and self-complementarity to ensure specificity and efficiency. Default parameters were used for the primer design.

### 2.4. Sample Selection and PCR Amplification

For SSR polymorphism testing, eight DNA samples were selected to represent all three geographic collection sites (Sera Temple: n = 2, Zhigongti Temple: n = 3, Kangba Temple: n = 3). These individuals were randomly chosen from the 52 successfully extracted samples to ensure unbiased representation of the study population. Polymorphism of the candidate primers was evaluated using these samples. Two-stage PCR amplification was performed as follows: The initial amplification utilized forward primers concatenated with universal 16-base-tag sequences in a 20 μL reaction mixture containing 17 μL TSINGKE Green Taq Mix, 1 μL each of 10 μM forward and reverse primers, and 1 μL genomic DNA template. Thermal cycling conditions comprised an initial denaturation at 98 °C for 2 min, followed by 35 cycles of denaturation (98 °C, 10 s), annealing (60 °C, 10 s), and extension (72 °C, 10 s), with a final extension at 72 °C for 5 min. In the second round, PCR products with ideal amplification bands were screened for fluorescent primer PCR using tag-modified primers and corresponding reverse primers, following the same thermal cycling protocol as the first round. Amplification success was verified by electrophoresing 4 μL of PCR products on 2% agarose gels at 300 V for 12 min. Gel images were analyzed to estimate the DNA concentrations, which were then adjusted by diluting with sterile water to the required concentration for capillary electrophoresis.

### 2.5. Capillary Electrophoresis and Data Analysis

For fragment analysis, a master mix was prepared by combining HiDi formamide with GeneScan 500 LIZ Size Standard at a 130:1 volume ratio. Ten microliters of this mixture was dispensed into each well of a 96-well plate, followed by the addition of 0.5 μL fluorescent PCR product per sample. Following brief centrifugation at 4000 rpm to ensure homogeneity, the samples were denatured at 95 °C for 5 min and immediately snap-cooled at −20 °C. After thawing and vortexing, the processed samples underwent capillary electrophoresis on an ABI 3730xl Genetic Analyzer (Applied Biosystems, Foster City, CA, USA). Allele sizing and polymorphism assessment were performed using gene mapper 6 software.

## 3. Results

### 3.1. Characteristic of Microsatellite Loci in the Genome of G. himalayensis

SSRs can be classified as perfect, imperfect, or compound based on their structure. Perfect SSRs have uninterrupted repeating motifs, while imperfect SSRs contain point mutations. Compound SSRs consist of two or more repeating motifs. This study focused on perfect and imperfect SSRs. In general, repetitive motifs that are cyclic or reverse complementary are considered to be of the same motif type [26,27]. For example, ACG refers to ACG, CGA, GAC, CGT, GTC, and TCG. A total of 240,741 microsatellites were identified in the Himalayan griffon genome, with an average density of 202.2 SSRs per Mb and an average length of 21.9 bp. The total length of the microsatellite sequences was 5.27 Mb, accounting for 0.44% of the genome. Perfect microsatellites (83.2%) were significantly more abundant than imperfect ones (16.8%). Mononucleotide repeats (53.2%) were the most prevalent, followed by penta- (13.6%), tetra- (13.1%), tri- (10.5%), di- (6.9%), and hexanucleotide repeats (2.8%) (Table 1).

**Figure 1:**
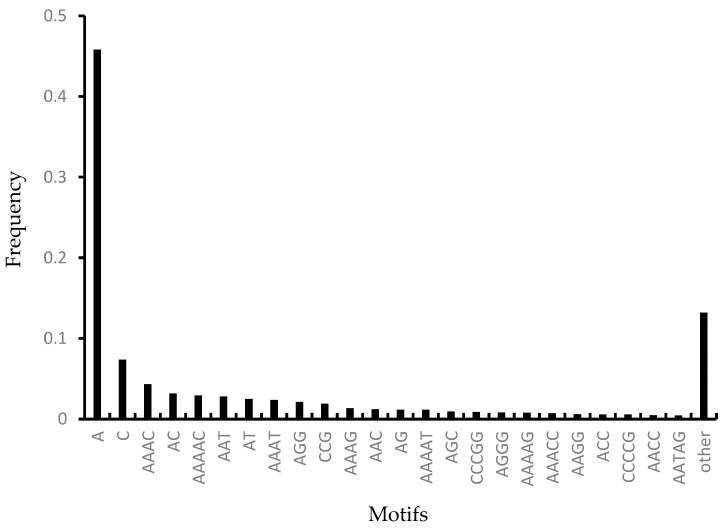
Frequency of microsatellites with different motifs in the genome of *G. himalayensis*.

Among the mononucleotide repeats, A and T repeats accounted for 86.1%. Among the dinucleotide repeats, AC had the largest number of microsatellites (7646); further, the AT motif also showed a significant numerical advantage (6010), followed by AG (2795), and CG had a very small number (83), similar to the microsatellite richness distribution pattern of most species. Among the trinucleotide repeats, the AAT motif (6729) was the dominant motif in the genome, followed by the other three relatively abundant repeats: AGG (5105), AAC (2917) and AGC (2274). These four types of repetitive motifs accounted for 67.6% of all trinucleotide motifs, and the remaining six types of trinucleotide repetitive motifs accounted for only 32.4% of microsatellites. Among the 32 tetranucleotide motifs, AAAC had the largest number of microsatellites (10,362), followed by AAAT (5658) and AAAG (3178), while AGGG (1973), AAGG (1385), and AACC (1107) had a similar number of microsatellites. These five types of microsatellites accounted for 75.1% of the tetranucleotide repeats. Among the 94 pentanucleotide repeat motifs, the microsatellites of the AAAAC motif accounted for 21.4% (7006), followed by AAAAT (27,818.5%), CCCGG (20,716.3%), AAAAG (18,975.8%), AAACC (17,385.3%), CCCCG (13,574.1%), and AATAG (10,643.2%). Among the 224 hexanucleotide repeat motifs, no microsatellite with a repeating motif appeared more than 1000 times in the genome, and the motif types with more than 500 occurrences were CCCCGG (657) and AAAAAG (527). The other 222 motif types were very rare. The order of the numerically dominant motifs is shown in Figure 1. The 24 microsatellite types that were detected more than 1000 times in the genome of Himalayan Griffon accounted for 86.8% of the total number of microsatellites. A/T appeared most frequently (45.8%), followed by C/G (7.4%) and AAAC/TTTG (4.3%) (Figure 1).

An analysis of the *G. himalayensis* genome revealed 24 microsatellite types with over 1000 occurrences, collectively representing 86.8% of all microsatellites. Among these, A/T repeats were the most abundant (45.8%), followed by C/G (7.4%) and AAAC/TTTG (4.3%) (Figure 1). Perfect microsatellites were more prevalent than imperfect ones across all motif types. Statistical analysis of repeat counts indicated that 69.8% of these abundant motifs had fewer than 20 repeats, while only 5.7% exhibited higher repeat numbers (≥30) (Table S1). Specifically, for microsatellites with more than 20 repeats, mononucleotide SSRs were the most frequent, with 61,847 occurrences, accounting for 48.3% of all mononucleotide microsatellites. In contrast, dinucleotides had 822 (5.0%), trinucleotides contained 313 (1.2%), tetranucleotides showed 78 (0.3%), and pentanucleotides presented 120 (0.4%). The majority of microsatellite loci exhibited low repeat numbers, particularly for tri-, tetra-, and pentanucleotide repeats, which predominantly ranged between 5–10 repeats.

**Figure 2:**
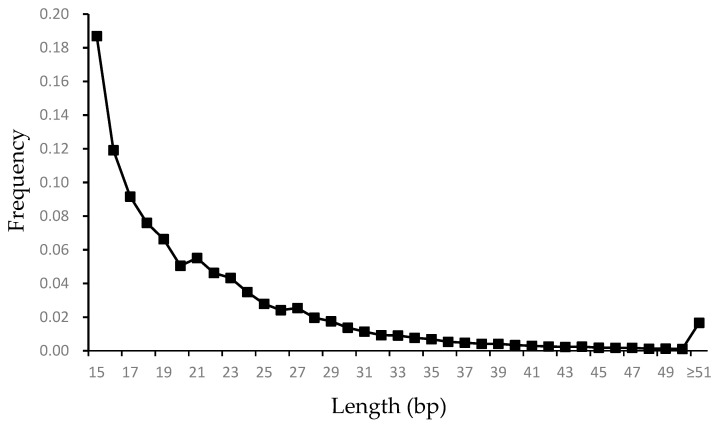
Frequency of microsatellites with different lengths in the genome of *G. himalayensis*.

Under the identification criteria used in this study, microsatellites with a length of 15 bp were found to be the most dominant, accounting for 18.7% (44,991) of all microsatellites. Among these, mononucleotide microsatellites were the most abundant (19,667), followed by pentanucleotide (8686), tetranucleotide (9666), trinucleotide (4399), and dinucleotide microsatellites (2573). The relationship between microsatellite length and frequency is illustrated in Figure 2. As the figure demonstrates, the frequency of microsatellites in the genome decreased as their length increased.

### 3.2. Design and Evaluation of SSR Primers

**Figure 3:**
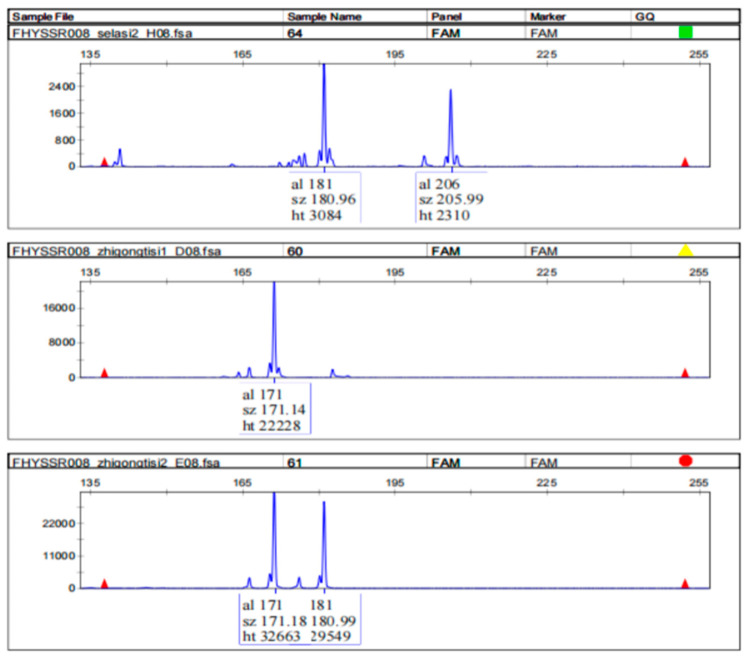
Analysis of capillary electrophoresis detection of samples with the FHYSSR008 primer.

A total of 100 primer pairs were designed based on the flanking sequences of the identified microsatellites. Loci exhibiting a high specificity and strong polymorphism were selected for further analysis (e.g., FHYSSR008, Figure 3). Based on the results, the polymorphic nature of fragments amplified by different primers was determined. After rigorous screening, 17 primers (Table 2) with a good polymorphism and high specificity were selected.

## 4. Discussion

The present study provides a comprehensive analysis of microsatellite characteristics in the genome of *G. himalayensis* and successfully develops a set of species-specific SSR primers. These findings offer valuable genetic tools and insights for further research on this ecologically important species.

Microsatellites constituted approximately 0.44% of the *G. himalayensis* genome, a coverage level consistent with that observed in other bird species [28], but significantly lower than that of humans (3%) [29], primates (0.83–0.88%) [30,31,32], cows (4.78%) and sheep (4.8%) [33], mice (2.85%) [34], and platypus (2.67%) [35], which directly validates Primmer et al.’s hypothesis [36] of reduced microsatellite abundance in birds relative to mammals. This scarcity of microsatellites in bird genomes may be attributed to their relatively small genome sizes [37,38], as microsatellite abundance is positively correlated with genome size [39,40,41]. Furthermore, the absence of poly(A) tails in SINE/LINE elements in birds, unlike mammals, may also contribute to this pattern, as poly(A) tails are recognized as a source for the evolution of simple repeats [36,42,43,44].

The predominance of mononucleotide SSRs in the *G. himalayensis* genome reflects a conserved evolutionary pattern across eukaryotes, with particularly strong convergence in avian and mammalian lineages [14,31,44,45,46]. This phenomenon is mechanistically rooted in the thermodynamics of DNA replication: shorter motifs (e.g., mononucleotides) require lower de-chain energy to form transient single-stranded regions during replication [47], enabling more frequent strand misalignment and slippage events per unit length [48]. Consequently, replication error rates exhibit a negative correlation with motif length, as evidenced by the higher mutation rates of mononucleotide SSRs in mammalian cells, such as those of humans [49,50]. These biophysical constraints create an evolutionary feedback loop—heightened slippage propensity not only accelerates SSR expansion but also ensures the preferential retention of short motifs through their elevated mutation-selection balance. Notably, while motifs longer than 3 bp face dual selective pressures (reduced slippage rates and energetic penalties in secondary structure formation [47]), tetranucleotide SSRs emerge as an exception in avian genomes, showing comparable abundance to pentanucleotide types and significant enrichment over di-, tri-, and hexanucleotide repeats—a pattern mirroring the tetranucleotide bias observed across birds [28].

The non-random distribution of microsatellites in the Himalayan griffon genome reveals two evolutionarily conserved principles governing SSR dynamics. First, the predominance of AT-rich motifs (e.g., mononucleotide A/T and tetranucleotide AAAC) mirrors patterns seen across distantly related taxa, including primates, rodents, and arthropods [42,51,52], suggesting universal biophysical constraints during replication. This AT bias arises from the thermodynamic instability of A/T base pairing—their weaker bond energy between A and T compared to G and C reduces the energy barrier for strand separation, facilitating higher slippage rates during replication [53,54]. Second, GC-rich SSRs face dual evolutionary disadvantages: stronger base pairing not only suppresses replication slippage, but also promotes stable secondary structures that are targets of mismatch repair systems [55], collectively limiting their genomic persistence. Understanding these patterns is essential for interpreting genetic variation and population structure, as they provide insights into the evolutionary dynamics of microsatellites and their role in shaping genomic diversity.

The analysis of repeat counts and microsatellite length distribution in the *G. himalayensis* genome revealed conserved eukaryotic patterns [42,56,57,58], with 69.8% of motifs containing fewer than 20 repeats and only 5.7% exhibiting ≥30 repeats, demonstrating the genomic dominance of short microsatellites. This trend is consistent with the hypothesis that microsatellites with lower repeat numbers are more stable and less prone to mutations, whereas longer repeats are subject to stronger selective pressures or higher mutation rates that limit their expansion [59,60]. Notably, mononucleotide repeats dominated among microsatellites with higher repeat numbers, accounting for 48.3% of all mononucleotide SSRs with more than 20 repeats. In contrast, di-, tri-, tetra-, and pentanucleotide repeats with higher repeat numbers were significantly less abundant, further supporting the notion that longer motifs are more susceptible to instability and are, therefore, less common in the genome [48]. Additionally, the observed decrease in microsatellite frequency with increasing length further supports the hypothesis that longer repeats are less stable and more prone to deletions or mutations [61,62].

The development of 100 primer pairs and the subsequent identification of 17 highly polymorphic and specific primers represent a significant milestone in genetic research on the Himalayan griffon. These primers offer versatile applications in various genetic studies of this species. In population genetics, they can be utilized to assess genetic diversity, population structure, and gene flow among different populations [63]. By genotyping individuals from diverse regions, researchers can gain critical insights into the genetic connectivity and differentiation of the species, which are essential for designing effective conservation strategies [64,65]. For instance, if specific populations exhibit low genetic diversity or significant genetic differentiation, targeted conservation measures—such as habitat protection and captive breeding programs—can be implemented to preserve the genetic viability of the species. Meanwhile, SSR markers can help construct more accurate phylogenetic trees by comparing microsatellite profiles across related species [66,67], which is invaluable for understanding the ecological niche and adaptive evolution of the Himalayan griffon within the broader avian family. Overall, these primers provide a powerful toolset for advancing both conservation and evolutionary research on this ecologically significant species.

## 5. Conclusions

This study provides the first comprehensive genome-wide characterization of microsatellites in the Himalayan griffon, identifying 240,741 SSRs with an average density of 202.2 SSRs per Mb, accounting for 0.44% of the genome. Mononucleotide repeats were the most abundant, followed by penta- and tetranucleotide repeats. A total of 100 primer pairs were designed, with 17 demonstrating high polymorphism, offering valuable tools for genetic diversity studies. These findings lay a crucial foundation for future research on the genetic structure, population dynamics, and conservation strategies of this near-threatened species. The developed SSR markers will facilitate population genetics, phylogenetic studies, and conservation efforts, contributing to the preservation of the Himalayan griffon and its ecological role as a scavenger in the Qinghai–Tibet Plateau. Continued efforts to refine and expand these genetic tools are essential for advancing our understanding and conservation of this ecologically significant species.

## Figures and Tables

**Table 1:** The number, proportion, average length, average mismatches and density of SSRs with different motif length in the genome of *G. himalayensis*.

Motif	Number of SSRs	Proportion (%)	Average Length (bp)	Average Mismatches	Counts/Mbp
Mononucleotide	128,024	53.18	20.98	0.21	107.52
Dinucleotide	16,534	6.87	22.91	0.38	13.89
Trinucleotide	25,186	10.46	23.67	0.56	21.15
Tetranucleotide	31,514	13.09	19.93	0.28	26.47
Pentanucleotide	32,804	13.63	23.97	0.31	27.55
Hexanucleotide	6679	2.77	29.85	0.69	5.61

**Table 2:** Primer and polymorphic information of the 17 SSR markers.

Marker ID	Repeat	* Primer Sequences (5′ → 3′)	Alleles	Size (bp)	PIC ^#^	Heterozygosity	
						Expected (He)	Observed (Ho)
FHYSSR008	(aggag)13	F -tatttccaggtgcagggacc R -tccctctctcatgtcccagg	8	161–206	0.817	0.836	0.5
FHYSSR009	(ccggc)4	F -ctcgtttgctgatgcctgc R -cccaccacgttacctgagc	5	168–188	0.717	0.758	0.75
FHYSSR012	(cctct)5	F -gacagctctggatcagctcc R -ctgtcaacaaaacccgtccc	3	174–184	0.427	0.477	0.625
FHYSSR016	(ggctc)4	F -ggtcagttgtccgcgatcc R -gaggaaccgacccaaccc	4	143–174	0.53	0.609	0.75
FHYSSR017	(gccga)8	F -ggtctgtgcaccctgagg R -atgcaaggagaggggaggg	4	230–250	0.458	0.492	0.375
FHYSSR024	(gtttg)13	F -atccaggtttgtcttgggcg R -tgctggctgcttatttcaagc	4	205–222	0.644	0.695	0.625
FHYSSR037	(tg)8	F -gttaaggactctgatgctgatgc R -acattagacgtgcagcgacc	3	147–152	0.294	0.32	0.375
FHYSSR043	(ca)10	F -ggtgaccacgtcttactcgg R -gctgtgcaccttgtctttcc	3	177–183	0.294	0.32	0.375
FHYSSR044	(at)11	F -acaatttcaaggctgcaatgc R -gtctccttcagcatccctcc	3	206–212	0.428	0.508	0.125
FHYSSR045	(tg)12	F -tcaggatccaacacagtgagc R -ctccctagtgcatcacgtgg	4	187–193	0.458	0.492	0.375
FHYSSR046	(tc)7	F -cgcctaccctaccccacc R -tgtgcaagtgattaagggattgg	2	168–170	0.258	0.305	0.125
FHYSSR048	(tat)5	F -cgtttcctgttcacacaagcc R -tgcttaaaacactggacacgc	2	179–188	0.375	0.5	0.25
FHYSSR059	(gct)5	F -gttcctcagaccgtctctgg R -atgtcagaagctcacctgcg	3	180–189	0.521	0.586	0.5
FHYSSR063	(gag)8	F -gaaaggttccccggaccg R -ctgcccaagtcctccagc	2	178–181	0.384	0.43	0.375
FHYSSR066	(ttc)9	F -cagttgttgctgatcgctgg R -gtcctcttctgcagggtgc	2	181–184	0.371	0.492	0.625
FHYSSR074	(act)6	F -ccccttcttaccttagtgacttcc R -cgactcatagctcttctagtggg	3	183–189	0.503	0.594	0.75
FHYSSR095	(tttg)5	F -tctggcattgtataccgggc R -tgtcccaaaggtcctttcagg	2	149–153	0.337	0.43	0.375

* F = forward; R = reverse. ^#^ Polymorphism information content.

## Data Availability

The data presented in this study are available in the article.

## Supplementary Materials

The following supporting information can be downloaded at: https://www.mdpi.com/article/10.3390/ani15101438/s1, Table S1. Number of repeats of the most frequent microsatellites in the genome of *G. himalayensis.*
