# Supplementary material for: Genome-Wide Microsatellite Characterization and Molecular Marker Development of Himalayan Griffon (Gyps himalayensis)
Source: Animals (Basel). 2025 May 16;15(10):1438. doi: 10.3390/ani15101438 (PMC12108375; doi:10.3390/ani15101438)
Supplement: Supplementary file 1 [file animals-15-01438-s001.zip › animals-3595690-supplementary.pdf]

Table S1. Number of repeats of the most frequent microsatellites in the genome of *G. himalayensis*.

	Iteration				Abundance and proportion （%）		
SSRs type	≤ 9	10–19	20–29	≥ 30	Total	Perfect SSRs	Imperfect SSRs
A	-	54730	44274	11275	110279 (45.81)	93650 (38.90)	16629 (6.91)
C	-	11447	5904	394	17745 (7.37)	16647 (6.91)	1098 (0.46)
AAAC	10306	55	-	1	10362 (4.30)	8962 (3.72)	1400 (0.58)
AC	3598	3722	303	23	7646 (3.18)	6227 (2.59)	1419 (0.59)
AAAAC	6986	19	1	-	7006 (2.91)	5871 (2.44)	1135 (0.47)
AAT	4435	2150	136	8	6729 (2.80)	3723 (1.55)	3006 (1.25)
AT	2286	3333	348	43	6010 (2.50)	4316 (1.79)	1694 (0.70)
AAAT	5570	88	-	-	5658 (2.35)	4884 (2.03)	774 (0.32)
AGG	4025	972	86	22	5105 (2.12)	3626 (1.51)	1479 (0.61)
CCG	4005	547	21	2	4575 (1.90)	3410 (1.42)	1165 (0.48)
AAAG	2884	253	33	8	3178 (1.32)	2169 (0.90)	1009 (0.42)
AAC	2716	193	8	-	2917 (1.21)	2536 (1.05)	381 (0.16)
AG	1563	1127	85	20	2795 (1.16)	2175 (0.90)	620 (0.26)
AAAAT	2697	83	1	-	2781 (1.16)	2259 (0.94)	522 (0.22)
AGC	2022	230	18	4	2274 (0.94)	1883 (0.78)	391 (0.16)
CCCGG	2064	7	-	-	2071 (0.86)	1814 (0.75)	257 (0.11)
AGGG	1915	53	5	-	1973 (0.82)	1473 (0.61)	500 (0.21)
AAAAG	1703	83	65	46	1897 (0.79)	1412 (0.59)	485 (0.20)
AAACC	1725	12	1	-	1738 (0.72)	1529 (0.64)	209 (0.09)
AAGG	1161	193	20	11	1385 (0.58)	915 (0.38)	470 (0.20)
ACC	1269	87	8	-	1364 (0.57)	1195 (0.50)	169 (0.07)
CCCCG	1335	22	-	-	1357 (0.56)	1086 (0.45)	271 (0.11)
AACC	1105	2	-	-	1107 (0.46)	1032 (0.43)	75 (0.03)
AATAG	887	171	6	-	1064 (0.44)	840 (0.35)	224 (0.09)
Total	66257	79579	51323	11857	209016 (86.82)	173634 (72.12)	35382 (14.70)
